# Overcoming barriers to COVID‐19 herd immunity in Afghanistan

**DOI:** 10.1002/puh2.161

**Published:** 2024-02-20

**Authors:** Mohammad Faisal Wardak, Ali Rahimi, Don Eliseo Lucero‐Prisno, Adriana Viola Miranda

**Affiliations:** ^1^ Scientific Research Center Jami University Herat Afghanistan; ^2^ Faculty of Medicine Jami University Herat Afghanistan; ^3^ Department of Global Health and Development London School of Hygiene and Tropical Medicine London UK; ^4^ Faculty of Management and Development Studies University of the Philippines Open University Los Baños Laguna Philippines; ^5^ Faculty of Public Health Mahidol University Bangkok Thailand; ^6^ Faculty of Medicine Universitas Indonesia Jakarta Indonesia

**Keywords:** Afghanistan, COVID‐19, herd immunity, pandemic, vaccination

## Abstract

Afghanistan faces numerous obstacles in its endeavor to achieve herd immunity against COVID‐19. Inadequate resources, vaccine hesitancy, and the new government's lack of international recognition have impeded the country and harmed vaccine procurement and distribution. Although the recent power shift has led to a more secure situation, the country's vaccination coverage remains at 44.05% as of November 26, 2023. The main hurdles to herd immunity include limited vaccine accessibility, extensive vaccine hesitancy, an inefficient cold chain system causing high vaccine wastage rates, substandard service delivery, recent restrictions on women's healthcare access, low health literacy, and a weakened economy owing to decades of conflict and international sanctions. This article assesses vaccination progress, and herd immunity barriers, and provides solutions to overcome them in Afghanistan. A comprehensive approach is required, which involves enhancing public awareness of the benefits of vaccination, debunking vaccine‐related misconceptions through the media, expanding vaccine accessibility especially in remote areas, increasing vaccination personnel, promoting the cold chain and delivery system, reversing the ban on women's education and employment, boosting the economy, and increasing the inflow of humanitarian aid by lifting international sanctions. Successfully implementing these measures can help Afghanistan overcome obstacles to herd immunity, leading the country toward a safer and healthier future.

## INTRODUCTION

The COVID‐19 pandemic had a widespread impact on the world since its emergence in December 2019, resulting in millions of cases and deaths globally. The pandemic also had a major impact on the economy, leading to job losses, a decline in per capita income, food insecurity, and decreased agricultural production [1]. As of December 6, 2023, Afghanistan has reported 229,263 confirmed cases and 7965 confirmed deaths. However, these figures are likely greatly underestimated due to restricted testing capabilities and inadequate reporting on fatalities. Estimates suggest that the actual number of deaths may range from 60,000 to nearly 200,000 [7]. Afghanistan has experienced four waves of COVID‐19 since its initial case in 2020, with the third wave having the greatest impact. The third wave reached its peak in June and July of 2021 as the delta variant of COVID‐19 started to spread rapidly in an unprecedented manner among the Afghan population in these 2 months. The number of confirmed cases and deaths more than doubled in these months from 72,977 cases and 2973 deaths to 147,501 cases and 6737 deaths, respectively. The onset of the COVID‐19 outbreak placed an immense strain on the healthcare system, which was already grappling with challenges such as limited resources, inadequate coverage, and funding constraints [2, 3].

The Western‐imposed sanctions on the Taliban‐led Afghan government have exacerbated the already dire hardships faced by the country's health sector. International experts warn that large portions of Afghanistan's healthcare system are teetering on the brink of collapse. Since the Taliban's takeover and the suspension of international aid payments to Afghanistan by the World Bank in August 2021, the nation has been grappling with a deepening humanitarian crisis, marked by famine and economic collapse [4]. The economy has declined by 30%, causing a loss of 700,000 jobs, and food insecurity affects nearly 90% of the population [5]. This crisis has left many medical professionals without pay for months, and health facilities are severely lacking even the most essential supplies for patient care. As a result, critical healthcare services, including the response to the ongoing COVID‐19 pandemic, have been severely disrupted [4].

Herd immunity, achieved when a significant portion of a population is immune to a virus through vaccination or prior infection, is crucial for controlling the spread of COVID‐19 [6]. Vaccination stands out as a reliable and secure approach for achieving herd immunity, concurrently reducing both mortality and morbidity. The effectiveness of this method can be further enhanced through the administration of booster doses, providing an added layer of immunity. The threshold for achieving herd immunity is contingent on the specific virus, and in the case of COVID‐19, it is determined by the proportion of the population immunized, represented as 1 − (1/*R*_0_). With the *R*_0_ (basic reproduction number) for SARS‐CoV‐2 ranging from 1.9 to 6.5, the corresponding herd immunity threshold falls within the range of 48%–85%. This implies that to curb the spread of the virus effectively, a significant percentage of the population needs to have immunity either through the vaccine or prior infection [6, 7].

Afghanistan has a population of 41 million people, and herd immunity requires between 19.3 and 35.7 million people to be immune against the virus. However, only 44.05% of the population (18 million) has been fully vaccinated as of November 26, 2023 [3]. The significance of individuals who have been previously infected with COVID‐19 in achieving herd immunity should not be understated. Although the reported confirmed cases stand at 200,000‐plus, the actual number is likely significantly higher due to underreporting and limited testing capacities. Nevertheless, those who have recovered from the virus contribute to the overall immunity in the population. The immunity gained through natural infection, coupled with vaccinations, collectively moves the nation closer to the desired herd immunity threshold. Moreover, with 18 million people already vaccinated, the country is on the verge of reaching the lower end of the herd immunity threshold (19.3 million). The ongoing progress is significant, showing that, despite the geopolitical complexities, the effort to vaccinate the Afghan population is moving forward. This ignites a hope that persisting with the vaccination campaign, despite its challenges, will lead to achieving herd immunity. This, in turn, stands to make a meaningful contribution to reducing the impact of COVID‐19 in Afghanistan. Our commentary addresses the current vaccination status, challenges, and recommendations for achieving herd immunity in Afghanistan.

## VACCINATION CAMPAIGN DURING TALIBAN RULE

The Taliban opposed immunization programs before taking power, claiming that they were a Western conspiracy to sterilize Muslims, or that they were a cover to target people [8]. They even issued fatwas (religious decrees) against polio vaccination and targeted vaccine officials. However, their stance has undergone a shift, and they have begun to take tentative steps in implementing measures to curb the spread of COVID‐19. This change in position may be due to the virus spreading among their ranks, and it could be part of their larger strategy to gain acceptance as responsible actors [8]. Despite the Taliban's history of disrupting public health efforts, their takeover in August 2021 brought an end to the conflicts that had sabotaged the vaccination campaign, leading to more effective vaccination efforts by the World Health Organization (WHO), United Nations Children's Fund (UNICEF), and other affiliated nongovernmental organizations (NGOs) [5]. Although the country's overall security situation has improved, violence remains an issue, particularly as the Islamic State Khorasan Province (ISKP) has increased attacks on civilians throughout the country [9]. It may create significant challenges in ensuring secure and accessible vaccination.

It should be noted that although the Taliban now cooperates with NGOs in the vaccination efforts and takes the security of vaccinators seriously, the vaccination campaigns remain under the full control of the NGOs rather than the Taliban government. The COVID‐19 vaccination program is now implemented independently of the government by the WHO, UNICEF, and related NGOs to accelerate the procedure [10]. Two main vaccines are currently in use in Afghanistan: Janssen Jcovden (single‐dose only) and Indian Covishield (Oxford/AstraZeneca formulation), and there is no booster dose vaccination plan [2].

**FIGURE 1:**
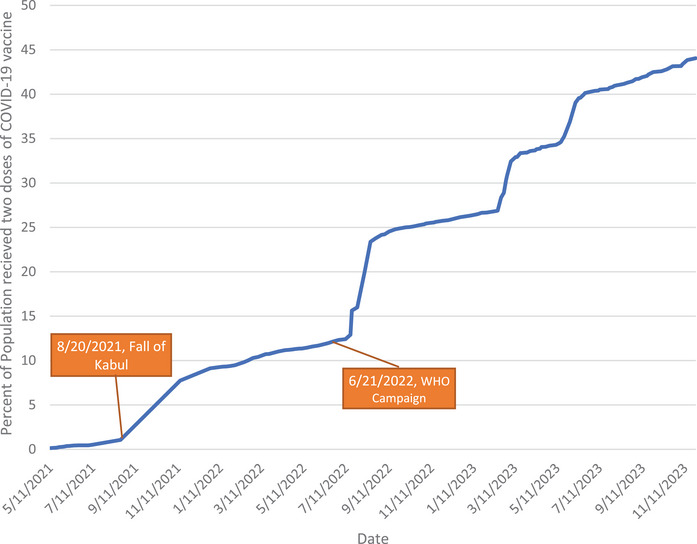
Share of people (%) who received two doses of the COVID‐19 vaccine in Afghanistan as of November 26, 2023 [3].

The vaccination rate in Afghanistan picked up pace in two timeframes. Before August 2021, the increase was gradual due to political instability, widespread security concerns, and a shortage of vaccines [2]. The first acceleration occurred after the Taliban took over and improved security. The second acceleration was due to a WHO campaign in June 2022 [10] (Figure 1).

## CHALLENGES TO ATTAINING HERD IMMUNITY

The implications of Afghanistan's failure to achieve COVID‐19 herd immunity extend beyond the borders of the country and have significant global consequences. Afghanistan's failure to reach herd immunity leaves a vulnerable population susceptible to the continued spread and mutation of the virus. This not only poses a continued threat to the health and well‐being of the Afghan people but also increases the risk of exporting new variants of the virus to neighboring regions and the world at large. Given the interconnectedness of the global community, the persistence of COVID‐19 in Afghanistan could hinder efforts to control the pandemic worldwide.

The COVID‐19 pandemic has posed several challenges to Afghanistan in its efforts to achieve herd immunity. Access to vaccination sites has been a challenge, particularly in remote areas, owing to geographical barriers [2]. In addition, there is a shortage of healthcare personnel, particularly female personnel, in remote areas. According to the current government guidelines and cultural norms, only female vaccinators are permitted to vaccinate women. Moreover, the lack of infrastructure, such as roads and transportation systems, in some regions of the country has made it difficult to transport and distribute vaccines effectively. Additionally, marginalized populations like Kuchis and internally displaced persons (IDPs) face additional barriers to vaccine access due to limited healthcare facilities and information.

Another significant challenge is the lack of an efficient cold chain system and service delivery, which has resulted in high rates of vaccine wastage [11]. Currently, there are no national cold chain maintenance plans or skilled technicians in Afghanistan [12]. The transportation of vaccines also faces basic challenges, such as a lack of refrigerated vaccine vans and freelance drivers with insufficient information and skills for the sensitive transportation of vaccines. Other issues include insufficient cold chain space, unreliable electricity supply, lack of spare parts, and poor documentation of repair dates [11]. This has resulted in Afghanistan not receiving vaccines that require an ultracold chain (e.g., −70°C) and only being able to store vaccines up to −20°C at the national, regional, and provincial levels [11, 12].

Vaccine hesitancy has emerged as another major challenge in achieving herd immunity in Afghanistan, with many people refusing to be vaccinated due to misconceptions and misinformation about vaccines [13]. Cultural beliefs, illiteracy, and lack of knowledge about vaccines have also impeded the progress of nationwide vaccination efforts [2]. Furthermore, the absence of trust in the healthcare system exacerbates this issue. Previously, conspiracy theories and propaganda campaigns have led to unfounded claims that the polio vaccine was contaminated with substances such as pig blood, further eroding public trust in vaccines [8]. According to one study, many Afghans were reluctant to receive vaccines due to concerns about safety and quality or because they believed they already had sufficient natural immunity [13]. These challenges pose a significant threat to efforts to control the spread of the virus and to achieve herd immunity through vaccination.

The ascent of the Taliban to power has resulted in a series of adverse consequences for Afghanistan, including global sanctions, the freezing of foreign exchange reserves, and the international community's refusal to recognize them as a legitimate government [5]. These recent developments have resulted in a deeper economic downturn, causing healthcare professionals to either not receive payments on time or not receive them at all. This has made it challenging to retain them, further worsening the shortage of staff. The imposed sanctions have also impeded the process of procuring and distributing vaccines and medical supplies throughout the country, posing a significant obstacle to achieving herd immunity.

The Taliban issued a ban on girls and women attending high schools and universities and working in NGOs and governmental offices, with the only exception being in the health sector [5]. These restrictions have a significant impact on the country's COVID‐19 vaccination campaign and the herd immunity of its population. The Taliban's adherence to a fundamentalist interpretation of Islamic jurisprudence severely restricts women's freedom of movement and access to healthcare, education, and employment [8]. These measures severely restrict women's access to vaccinations and contribute to increased vaccine hesitancy. Humanitarian aid groups face a dilemma: Either suspending all aid deliveries until the ban is lifted or working within the terms of the restriction, risking sending a message of weakness to the caretaker government and showing approval of the bans.

## RECOMMENDATIONS

Several recommendations can be made to overcome the challenges faced in achieving herd immunity in Afghanistan.

### Improving vaccination access

To enhance access to vaccinations, it is crucial to engage the local population in a widespread manner. Addressing the barriers posed by geographical distance requires increasing the number of stationary vaccination sites and mobile vaccination teams. Improving women's access to vaccination requires hiring more female vaccinators and implementing female‐focused awareness initiatives. Efforts should be made to lift restrictions on women, particularly the ban on traveling without male chaperones. One possible way to enhance women's vaccination access is to enlist and educate vaccine advocates who are respected in the local community. They can help women overcome travel difficulties by offering transportation or accompaniment services to vaccination sites or by organizing home visits or drive‐through vaccinations. Extending vaccination services to Kuchi and IDP camps will require innovative strategies such as on‐site vaccination stations or transportation to nearby centers.

### Improving the cold chain system and the immunization supply chain

A robust and sustainable cold chain system is crucial to ensure that vaccines are stored and transported safely. This can be achieved through the development of a national cold chain maintenance plan, including transportation standards, capacity building for staff, and increasing the number of health facilities capable of providing immunization services. Improving the capacity of staff and technicians to properly administer vaccines is also crucial. Clear information on vaccine distribution and administration should be provided to promote transparency and accountability during the vaccination process. Regular temperature monitoring records should be kept and reviewed by supervisors at the vaccination sites. The private sector could provide resources, such as refrigerated vans and equipment. Efforts should be made to secure funding and technical support from international organizations and partners to improve the cold chain system.

### Combating vaccine hesitancy

The government needs to launch an extensive education campaign to address vaccine hesitancy by highlighting safety and effectiveness. It should implement communication campaigns to raise public awareness about the importance of vaccinations, with targeted messages to address misconceptions among vulnerable groups like women and marginalized communities. This should include targeted messages that address prevalent misconceptions and concerns regarding vaccines. The government should collaborate with healthcare providers, religious leaders, and community members to build trust and support vaccination. Lifting restrictions on women's education is crucial to address vaccine hesitancy among the female population. Leveraging social media and digital platforms is important to reach younger generations influenced by online misinformation about vaccines. Learning from successful initiatives in Bolivia, where community‐led pro‐vaccine mobilizations significantly boosted childhood vaccine acceptance, underscores the impact of local leaders. Additionally, intercultural knowledge dialogs, held in collaboration with radio stations, openly addressed COVID‐19 vaccination fears, reaching 200,000 individuals [14]. Collaborating with reputable sources like the WHO to share reliable and updated vaccine information is essential. The government should also encourage celebrities and social media influencers to promote and endorse vaccination among their followers.

### Lifting international sanctions

The Taliban must engage in a process of negotiation with the international community on matters concerning human rights, with a specific emphasis on women's and girls’ rights, and work toward reaching a mutually acceptable resolution. Lifting international sanctions would result in a significant increase in international aid, thereby improving the logistical framework for vaccine distribution. Releasing Afghanistan's frozen assets from foreign banks would allow investment in the healthcare system, including timely payments for healthcare workers and improved services. A stronger economy would improve overall health and contribute to herd immunity.

## CONCLUSION

Achieving herd immunity in Afghanistan is a complex challenge due to limited vaccine access, vaccine hesitancy, gender‐based restrictions on education and employment, inefficient cold chain and supply chain systems, and international sanctions, which restrict vaccine availability and distribution. To effectively tackle these hurdles, the Afghan government, NGOs, and the international community must work together to provide education, resources, and support to the Afghan population. It includes lifting international sanctions, expanding access to vaccines by increasing vaccination sites and personnel, raising awareness about the benefits of vaccination while dispelling myths and misinformation, improving the cold chain system and service delivery, and reversing restrictions placed on women. The success of this effort depends on the active cooperation of all stakeholders, including the Taliban‐led government, in partnership with WHO and other organizations involved in the vaccination campaign. Despite the challenges, WHO and affiliated NGOs have played a crucial role and demand continued support. Ultimately, the success of herd immunity hinges on a united and coordinated effort from all parties, including the active participation of the Afghan people.

## AUTHOR CONTRIBUTIONS

Don Eliseo Lucero‐Prisno III conceptualized the manuscript. Ali Rahimi and Mohammad Faisal Wardak wrote the original draft. Ali Rahimi, Mohammad Faisal Wardak, Don Eliseo Lucero‐Prisno III, and Adriana Viola Miranda reviewed and edited the manuscript.

## CONFLICT OF INTEREST STATEMENT

The authors have no conflicts of interest to declare for this study. Don Eliseo is Editor‐in‐Chief of the journal and co‐author of this article. Adriana Viola Miranda is an Editorial Board member of Public Health Challenges and a co‐author of this article. They were excluded from the peer‐review process and all editorial decisions related to the acceptance and publication of this article.
